# Ulceration of the Œsophagus in a Mule

**Published:** 1881-10

**Authors:** L. E. Wheat

**Affiliations:** D.V.S., Ont.; Scranton, Pa.


### V.—ULCERATION OF THE ŒSOPHAGUS IN A MULE.

Editor of Journal of Comparative Medicine:

In March, 1881, I was called by the Pennsylvania Coal Company to see a mule—the symptoms denoting general debility, staggering gait, difficult breathing, slavering from the mouth, difficulty in swallowing fluids, which were returned by the nose. The cellular tissue along the œsophagus being distended, I concluded I had a case of ruptured œsophagus to deal with. But upon inquiry, learned there had been no violence perpetrated; he had not choked, had worked well, and ate well until three days previous. Nothing was left now but to cut down upon this impacted mass and ascertain its source. I made a free incision posterior to thyroid glands—this being the most dependent part—when there escaped about three quarts of finely masticated hay and grain in a semi-fluid form. The surrounding tissues being so diseased, I dissected this away, exposing the œsophagus, where I found a fistula that would admit my three fingers. After dressing the edges, I closed it with silk sutures (having nothing better with me). I ordered gruel diet, the head to be tied to the rack, the wound dressed with borated cotton, saturated in carbolized oil, and retained by many-tailed bandage. They gave him bran mash, which, for four or five days, passed through the opening some; it eventually closing, giving but little trouble. I saw him about four weeks after; he was much improved, the diseased tissues being nearly reproduced by new.

L. E. Wheat, D.V.S., Ont.

Scranton, Pa.

